# SP-A2 contributes to miRNA-mediated sex differences in response to oxidative stress: pro-inflammatory, anti-apoptotic, and anti-oxidant pathways are involved

**DOI:** 10.1186/s13293-017-0158-2

**Published:** 2017-12-04

**Authors:** George T. Noutsios, Nithyananda Thorenoor, Xuesheng Zhang, David S. Phelps, Todd M. Umstead, Faryal Durrani, Joanna Floros

**Affiliations:** 1 Center for Host Defense, Inflammation, and Lung Disease (CHILD) Research, Department of Pediatrics, The Pennsylvania State University College of Medicine, Hershey, PA 17033-0850 USA; 2 Department of Obstetrics and Gynecology, The Pennsylvania State University College of Medicine, Hershey, PA 17033-0850 USA

**Keywords:** Surfactant protein A, Ozone, Sex differences, STAT3, IL-6

## Abstract

**Background:**

Human innate host defense molecules, surfactant protein A1 (SP-A1), and SP-A2 differentially affect the function and proteome of the alveolar macrophage (AM). We hypothesized that SP-A genes differentially regulate the AM miRNome.

**Methods:**

Humanized transgenic mice expressing SP-A1 and SP-A2 were subjected to O_3_-induced oxidative stress (OxS) or filtered air (FA), AMs were isolated, and miRNA levels were measured.

**Results:**

In SP-A2 males, we found significant changes in miRNome in terms of sex and sex-OxS effects, with 11 miRNAs differentially expressed under OxS. Their mRNA targets included BCL2, CAT, FOXO1, IL6, NF-kB, SOD2, and STAT3. We followed the expression of these transcripts as well as key cytokines, and we found that (a) the STAT3 mRNA significantly increased at 4 h post OxS and returned to baseline at 18 h post OxS. (b) The anti-oxidant protein SOD2 level significantly increased, but the CAT level did not change after 4 h post OxS compared to control. (c) The anti-apoptotic BCL2 mRNA increased significantly (18 h post OxS), but the levels of the other transcripts were decreased. The presence of the SP-A2 gene had a protective role in apoptosis of AMs under OxS compared to mice lacking SP-A (knockout, KO). (d) Pro-inflammatory cytokine IL-6 protein levels were significantly increased in SP-A2 mice compared to KO (4 and 18 h post OxS), which signifies the role of SP-A2 in pro-inflammatory protein expression. (e) SOD2 and CAT mRNAs changed significantly in OxS indicating a plausible role of SP-A2 in the homeostasis of reactive oxygen species. (f) Gonadectomy of transgenic mice showed that sex hormones contribute to significant changes of the miRNome expression.

**Conclusions:**

We conclude that SP-A2 influences the miRNA-mediated sex-specific differences in response to OxS. In males, these differences pertain to inflammatory, anti-apoptotic, and anti-oxidant pathways.

**Electronic supplementary material:**

The online version of this article (10.1186/s13293-017-0158-2) contains supplementary material, which is available to authorized users.

## Background

The major function of the lung is to enable the uptake of oxygen from ambient air and eliminate carbon dioxide from the circulation. As the lung performs this life-sustaining function, it encounters many insults from the environment, such as ozone (O_3_), particulate matter, allergens, bacteria, and viruses. Although healthy individuals bearing a well-regulated innate host defense system can neutralize these insults and maintain normal lung function, O_3_-induced oxidative stress (OxS) can have dire effects on pulmonary innate host defense mechanisms and consequently on lung function [1].

In the alveolus, the alveolar macrophages (AMs), and to a lesser extent, the alveolar epithelial cells provide the primary innate defense against inhaled insults that may trigger a cascade of inflammatory responses after O_3_ exposure. The alveolus is lined by epithelial type I and type II cells and contains various epithelial cell metabolic products, including pulmonary surfactant as well as proteins from the lung interstitial fluid. Both surfactant proteins and lipid constituents of surfactant influence the regulation and function of AM [2–9].

Surfactant protein A (SP-A) is an important host defense molecule and has both innate host defense functions [2, 10–12] and surfactant-related functions that influence surfactant secretion and structure. We and others have highlighted the function of SP-A as innate immune molecule itself by showing that SP-A knockout (KO) mice are more prone to several infections compared to mice that express SP-A [13–17]. Previous studies have described the regulatory effects of SP-A either by directly affecting AM function [2, 18], the proteomic expression profile of AM [9, 19], and the cell shape and activation state of AM [20] or indirectly via the production of cytokines and chemokines by AM [11, 21]. Ozone exposure induces production of reactive oxygen species (ROS) by bronchoalveolar cells [22]. ROS damage the lung epithelium [23] and oxidize SP-A, and in turn, this compromises its innate immune functions [24–29]. The model used in this study provides advantage over other types of OxS, such as hyperoxia (which occurs over days), as it produces effects in several hours, allowing us to examine primary endpoints rather than secondary downstream events.

Ozone exposure has resulted in significant sex differences in survival, with females showing higher morbidity and mortality rates than males in several lung diseases including respiratory infections, COPD, asthma, and CF [30–34]. In our studies, we observed differences in survival rates between males and females after bacterial infection. Females appeared to resolve the bacterial respiratory infection more readily than males, but when OxS was introduced prior to infection, the outcome was reversed, i.e., females showed lower survival rates than males [13, 17, 35–38]. Moreover, these sex differences were further accentuated in SP-A KO mice or when SP-A was oxidized, highlighting once again the important role of SP-A as a regulatory innate immune molecule [13, 17]. The observed sex differences are in part, if not entirely, attributable to circulating gonadal hormones, which are believed to influence the innate immune responses [36], although the specific roles of these hormones and the underlying mechanisms of regulation remain unknown.

In the present study, we hypothesized that the two human SP-A1 and SP-A2 gene products differentially regulate the AM miRNome and this explains in part the previously observed differences in AM function and the AM proteome. We exposed male and female mice expressing SP-A1 or SP-A2 to O_3_ or FA (control), and we measured the expression levels of 372 micro-RNAs (miRNAs). The miRNAs with significantly altered levels in response to OxS were identified and used for further analysis. The findings showed that the AM miRNome is regulated by O_3_ exposure and that the SP-A2 male miRNome is associated with genes involved in inflammation pathways, regulation of reactive oxygen species, and apoptosis. Moreover, the miRNA differences in SP-A2 male and female mice after gonadectomy showed that sex hormones play an important role in the observed miRNA differences by affecting the regulation of miRNAs. We postulate that miRNAs contribute to mechanisms involved in the regulation of AM function and the AM proteome by the SP-A2 gene in males.

## Methods

### Animals

We used humanized transgenic (hTG) C57BL/6 mice, expressing SP-A1 (6A^2^) or SP-A2 (1A^0^), or SP-A KO mice [39] at 12 weeks of age. Both males and synchronized females (regarding the estrous cycle) were used in this study. For synchronization, we used dirty bedding from a male’s cage to stimulate estrus in females. Females were group-housed on male bedding for exactly 10 days before exposure to FA or O_3_. For each experimental condition, there were 3 animals/group (*n* = 36 for miRNA study and *n* = 60 for gene expression analysis, terminal deoxynucleotidyl transferase (TdT)-mediated dUTP Nick-End Labeling (TUNEL) assay, and miRNA analysis in gonadectomized mice). A total of 96 animals were used for the entire study. All procedures involving animals used protocols that were approved by the Institutional Animal Care and Use Committee at the Pennsylvania State University College of Medicine. All mice were maintained in facilities under pathogen-free conditions or in barrier containment facilities.

### Ozone exposure

Mice were exposed to 2 ppm ozone (O_3_) or to filtered air (FA) (control) at room temperature and 50% humidity for 3 h, as described previously [40]. In all cases, we used at least 3 mice/group, i.e., 3 males, 3 females, 3 SP-A KO, 3 hTG SP-A2, 3 hTG SP-A1, 3 for O_3_ exposure and 3 for filtered air, and 3 male and 3 female SP-A2 and KO gonadectomized mice (*n* = 96). Each sample was analyzed individually, and we did not pool any samples. All FA and O_3_ exposures were conducted in parallel [41]. Mice were sacrificed 4 and 18 h after exposure.

### Mouse alveolar macrophage purification

Mouse AMs were obtained by bronchoalveolar lavage (BAL) [9]. AMs were separated from the BAL by centrifugation (150×*g* for 5 min). The BAL supernatant, which was cell-free (3.0 ml), was stored at − 80 °C until further use. The cell pellets were washed with 1× PBS (Gibco, Waltham, MA), and cells were counted. A fraction of the cells was placed in a cytocentrifuge and used to prepare cytospins, cells were stained, and a differential cell count was performed. AM purity was 95% as assessed by Papanikolaou staining. The remaining AM pellet was resuspended in 0.5 ml solution of 90% fetal bovine serum (Gibco) and 10% DMSO (Sigma-Aldrich, St. Louis, MO) and was cryopreserved in liquid nitrogen until further use.

### Isolation of miRNAs and qRT-PCR

Total RNA from the AM was isolated using QIAzol Lysis Reagent (#79306, Qiagen, Valencia, CA), and the miRNA-enriched fraction was isolated and purified with miRNeasy Micro Kit (#217084, Qiagen). cDNA was generated with the miScript II RT Kit (#218161, Qiagen) and then used as a template for real-time qPCR with the miScript SYBR Green PCR Kit (#218075, Qiagen). The expression profiles of the 372 most abundantly expressed and best-characterized miRNAs in miRBase were determined by miRNA PCR Array (Array #MIMM-3001Z, Qiagen). The miRNA PCR array covers most of the mouse miRNA orthologs (~ 90%) of the human miRNA annotations. The variability across the 3 samples is assessed by *p* values (*p* < 0.05). The miRNAs with significantly changed levels were studied further (*p* < 0.05).

### Data analysis

miRNAs (mmu-miR-21a-5p, mmu-miR-23a-3p, mmu-miR-2137, and mmu-let-7c-5p) were constant under our experimental conditions and therefore were selected for use in the normalization of all miRNA values. The arithmetic mean values of these miRNAs were used for normalization, and all statistical analyses were done after normalization.

The Ct cutoff was 35. The fold change (2^−ΔΔCt^) was calculated as the normalized individual miRNA expression (2^−ΔCt^) in each individual experimental specimen divided by the same normalized miRNA expression 2^−ΔCt^ in the corresponding KO (control). The fold regulation represented fold change results in a biologically meaningful way. Fold change values greater > 1 indicated a positive or an upregulation, and the fold regulation was equal to the fold change. Fold change values < 1 indicated a negative or downregulation, and the fold regulation was the negative inverse of the fold change. The *p* values were calculated based on Student’s *t* test of the replicate 2^−ΔCt^ values for each miRNA in the KO control group and treatment groups. All data can be found in Additional file 1. Data analysis was performed with manufacturer’s software (https://www.qiagen.com/us/resources/geneglobe/).

### Gonadectomy and ozone exposure

Male and female SP-A2 mice were gonadectomized (Gx) as described [36]. Two weeks after gonad removal, mice were exposed to O_3_ (2 ppm) for 3 h and were sacrificed 4 h post OxS. The RNA from AM was isolated, and the differential expression of miRNAs was quantified as tag count data [42] by RNA sequencing at the Pennsylvania State University College of Medicine Genomic Core Facility, with default false discovery rate 0.1 (cutoff). The miRNAs identified from Gx mice were selected based on the average tag count data (≥ 100) and with good correlation data count between mice (2 out of 3). We successfully identified 63 miRNAs in Gx-SP-A2 and KO (male and female) and used these in the present analysis. First, the changes in miRNA expression in SP-A2 mice were calculated by normalizing to KO, i.e., the level of expression of each individual experimental miRNA (i.e., in Gx-SP-A2) was divided by the same miRNA expression in the corresponding Gx-KO. Next, the differentially expressed miRNAs between SP-A2 males and females were determined by dividing a specific individual male miRNA by the corresponding specific female miRNA. Lastly, the fold changes of the identified miRNAs from Gx (SP-A2 male vs female) were compared to the corresponding miRNAs identified from non-gonadectomized (NGx) SP-A2 male vs female. Samples from 12 animals (6 males and 6 females for SP-A2 and KO) were individually analyzed.

### Gene expression assays

AMs were lysed by the addition of QIAzol Lysis Reagent (Qiagen). Total RNA was purified with Direct-zol RNA Mini Prep kit (#R2052, Zymo Research, Irvine, CA), and RNA concentration and quality were confirmed by Nanodrop and Bioanalyzer. RNA was reverse-transcribed using RT2 first strand kit (#330401, Qiagen), according to the manufacturer’s protocol. Real-time PCR was performed using RT2 SYBR Green ROX qPCR Mastermix (#330520, Qiagen) on a QuantStudio 12K Flex Real-Time PCR System (Applied Biosystems, Waltham, MA) at the Pennsylvania State University College of Medicine Genomic Core Facility. The expression levels of GAPDH, SOD2, CAT, IL-6, STAT3, BCL2, FOXO1, FOXO3, BECN1, IKKβ, and NF-kB-p65 were detected by qRT-PCR with specific RT2 qPCR Primer Assays (Qiagen). Samples from 3 animals/treatment (FA and O_3_) and 3 replicates/animal were individually analyzed and quantified relative to GAPDH mRNA expression. The relative expression levels of target genes were determined by the equation 2^−∆CT^, and 2^−∆∆CT^, in which ∆*C*_*T*_ was calculated as follows: ∆*C*_*T*_ = *C*_*T* gene-of-interest_ − *C*_*T* housekeeping gene_. The ∆∆*C*_*T*_ was calculated by the difference between ∆*C*_*T*_ values of experimental (O_3_ exposure) and control (FA exposure) (∆∆*C*_*T*_ = ∆*C*_*T* experimental_ − ∆*C*_*T* control_).

### Western blot analysis and antibodies

Equal amounts of AM protein lysates for each set of samples from control and O_3_ exposure mice were separated by sodium dodecyl sulfate-polyacrylamide gel electrophoresis (SDS-PAGE), transferred to PVDF membrane, and the expression levels of STAT3, NF-kB-p65, FOXO1, CAT, SOD2, and GAPDH were detected by Western blotting with specific antibodies. Samples from 3 animals/treatment (control and O_3_) were individually analyzed and quantified relative to GAPDH expression. The antibodies utilized were obtained from the following sources: NF-kB (#8242), FOXO1 (#2880), GAPDH (#2118), STAT3 (#12640), SOD2 (#13194), and catalase (#14097) (Cell Signaling Technology, Danvers, MA).

### Measurement of IL-6, TNF-α, and IL-1β

Data from BAL from 3 animals/treatment (FA, O_3_) and 3 replicates/animal were used to assess levels of IL-6, TNF-α, and IL-1β using a conventional enzyme-like immunosorbent assay (ELISA) as per vendor’s protocol (#SEM03015A, SEM03113A, and SEM03109A, SABiosciences, Valencia, CA). Levels of IL-6, TNF-α, and IL-1β were measured using BAL samples. (a) BAL from SP-A2 mice after 4 h of ozone (O_3_) exposure was used to measure IL-6 levels by diluting the BAL 1:2, 1:4, 1:8, and 1:16. (b) BAL from SP-A2 and KO mice after 18 h of O_3_ exposure was used to measure IL-6 levels by diluting 1:2, whereas the levels of TNF-α and IL-1β were measured without BAL dilution. The optical density of the end product was measured at 450 nm using Tecan Spectrafluor spectrophotometer (Agilent Technologies, Santa Clara, CA).

### TUNEL assay

The TdT-mediated dUTP Nick-End Labeling (TUNEL; #4822-96-K, HT Titer TACS Assay, Trevigen, Gaithersburg, MD) assay was used to detect the ability of ozone to induce apoptosis according to the vendor’s protocol. Briefly, AM cells (70,000 cells/well) after 18 h of O_3_ exposure from hTG SP-A2 and KO mice were fixed in 3.7% formaldehyde in 1× PBS. The cells were incubated for 1 h at 37 °C in the presence of TdT. In positive control TACS-Nuclease was used, whereas no TdT was added in the unlabeled control (data not shown). The apoptotic cell absorbance was measured at 450 nm using Tecan Spectrafluor spectrophotometer (Agilent Technologies).

### Statistical analysis

All experiments were done using 3 animals for each condition. Replicates were analyzed using Student’s *t* test; a *p* value of < 0.05 was considered statistically significant. Bonferroni correction of the *p* values was applied to account for the sex, treatment, and gene variability in our miRNA arrays. Therefore, we considered significant miRNA changes with *p* < 0.0166. Moreover, to control false discovery rates (FDR) within our miRNA arrays, we also determined the FDR by Benjamini and Hochberg approach [43]. We focused our analysis only on miRNAs passing both the Bonferroni correction and an FDR-adjusted threshold of 0.05 (expressed as *q* < 0.05) (see Additional file 1).

To report sex differences, a two-way analysis of variance (ANOVA) with factors of treatment and sex was performed to show a significant interaction between sex and treatment, meaning that the treatment effect differed in the two sexes (see Additional file 1). Also, the effect of gonadal removal in O_3_ response was assessed by one-way ANOVA (see Additional file 3).

## Results

### SP-A genes differentially regulate the AM miRNome

**Fig. 1:**
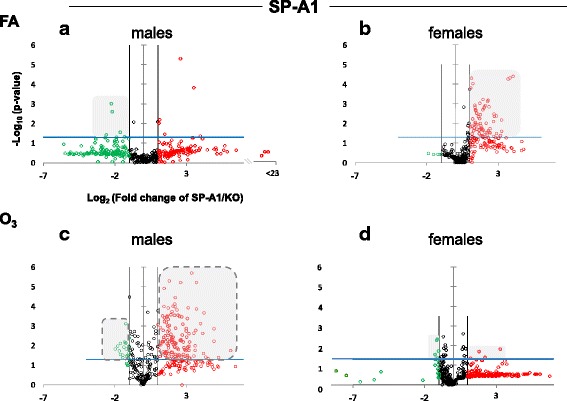
Volcano plots indicating the statistical significance of SP-A1 AM miRNome expression levels compared to SP-A KO under FA or O_3_ exposure for males and females. The *x*-axis plots the log_2_ of the fold changes, while the *y*-axis plots the −log_10_ of their *p* values based on *t* test of the replicate raw Ct data (see the “Methods” section). Each plot has 3 vertical lines. The middle vertical line that is graded corresponds to 0 changes. The lines on either side represent ≥ 2-fold differences. Dots in the volcano plots above the blue horizontal line identify fold changes with statistical significance of at least the Bonferroni corrected *p* < 0.0166. The red and green dots represent miRNAs that were upregulated ≥ 2-fold and downregulated ≤ − 2-fold, respectively, compared to KO. Black dots signify miRNAs that were regulated less than 2-fold times (i.e., − 2 ≤ *x* ≤ 2; *x* is the fold change). **a** Male SP-A1 mice compared to KO exposed to FA. **b** Female SP-A1 mice compared to KO exposed to FA. **c** Male SP-A1 mice compared to KO after O_3_ exposure. **d** Female SP-A1 mice compared to KO after O_3_ exposure. The shaded gray areas show the differences in the miRNAs that are highly and significantly regulated between the two sexes and the two conditions compared to KO. The shaded gray areas with a broken outline are to highlight the significantly changed miRNAs in SP-A1 male O_3_ exposed mice and SP-A2 male O_3_ exposed mice (Fig. 2). The *x*-axis on **a** is broken to allow comparison between all panels

**Fig. 2:**
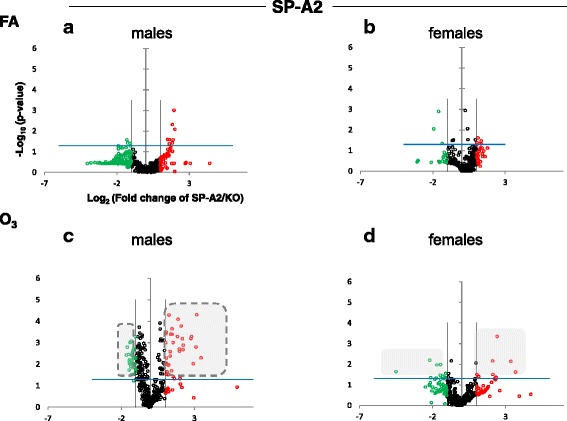
Volcano plots (as described in Fig. 1) indicating the statistical significance of SP-A2 AM miRNome expression levels compared to SP-A KO under FA or O_3_ exposure for males and females. **a** Male SP-A2 mice compared to KO exposed to FA. **b** Female SP-A2 mice compared to KO after FA exposure. **c** Male SP-A2 mice compared to KO after O_3_ exposure. **d** Female SP-A2 mice compared to KO after O_3_ exposure. The shaded gray areas with a broken outline compare the significantly changed miRNAs in SP-A2 male O_3_ exposed mice and SP-A1 male O_3_ exposed mice (Fig. 1)

The expression levels of the hTG SP-A1 and SP-A2 AM miRNomes were determined in males and females that were exposed to FA or O_3_ and compared to the corresponding KO AM. The miRNome levels are presented as volcano plots to show the fold change regulation differences between levels of miRNAs in hTG and KO mice, as well as their statistical significance (Figs. 1 and 2).

Under basal conditions, which in our experimental model is exposure to FA, panel A in Fig. 1 shows a very tightly packed cluster of data points with relatively few data points exceeding the cutoff for significance (Bonferroni corrected *p* < 0.0166), indicating that there are a few differences between FA-exposed SP-A1 males and KO males. In panel B, when the same comparison is made with female mice, we observe a multitude of miRNAs that are significantly upregulated. It is immediately obvious by comparing the shaded areas in panels A and B that there are many more differences (of both magnitude and significance) in females compared to males. Following O_3_ exposure (panels C and D), the SP-A1 mice show a very different picture. The female mice (panel D) exhibit fewer differences of lower magnitude than what is observed with the males (panel C). The pattern seen in OxS is almost the opposite of the basal conditions (compare shaded areas in Fig. 1).

When we compare the SP-A2 hTG males and females under basal conditions, we do not see any robust differences as with the SP-A1 hTG (Fig. 2, panels a and b, respectively). However, under OxS, females have less pronounced differences than males (Fig. 2, panels c and d). Under OxS, the male miRNome in both SP-A1 and SP-A2 mice is more responsive compared to females and exhibits a higher number of changing miRNAs that reach the Bonferroni corrected significance threshold *p* < 0.0166 (compare shaded areas). A two-way ANOVA test for the sex and treatment effects showed that the *F* stat for the SP-A2 mice regarding the sex effect is *F* = 26.34 with *F* crit = 3.84 and *p* = 3.23 × 10^−7^. The *F* stat for the interaction between the two factors (sex and treatment) was *F* = 25.25 with *F* crit = 3.84 and *p* = 5.65 × 10^−7^. The same analysis for the SP-A1 mice miRNome did not show that sex, treatment, or the combination of these two factors were significantly different (see Additional file 1). The ANOVA test for the SP-A1 mice to check the effect of treatment (FA or O_3_), sex (male, female), and the interaction between the two variables (treatment and sex effects) showed that the *F* stat for SP-A1 mice was *F* = 1.17 lower than the *F* crit = 3.85 and *p* value not significant *p* = 0.279. These data show that in the AM miRNome, there is a difference between sexes in response to O_3_ exposure with the presence of SP-A1 or SP-A2.

### Regulation of AM miRNome in response to OxS

**Fig. 3:**
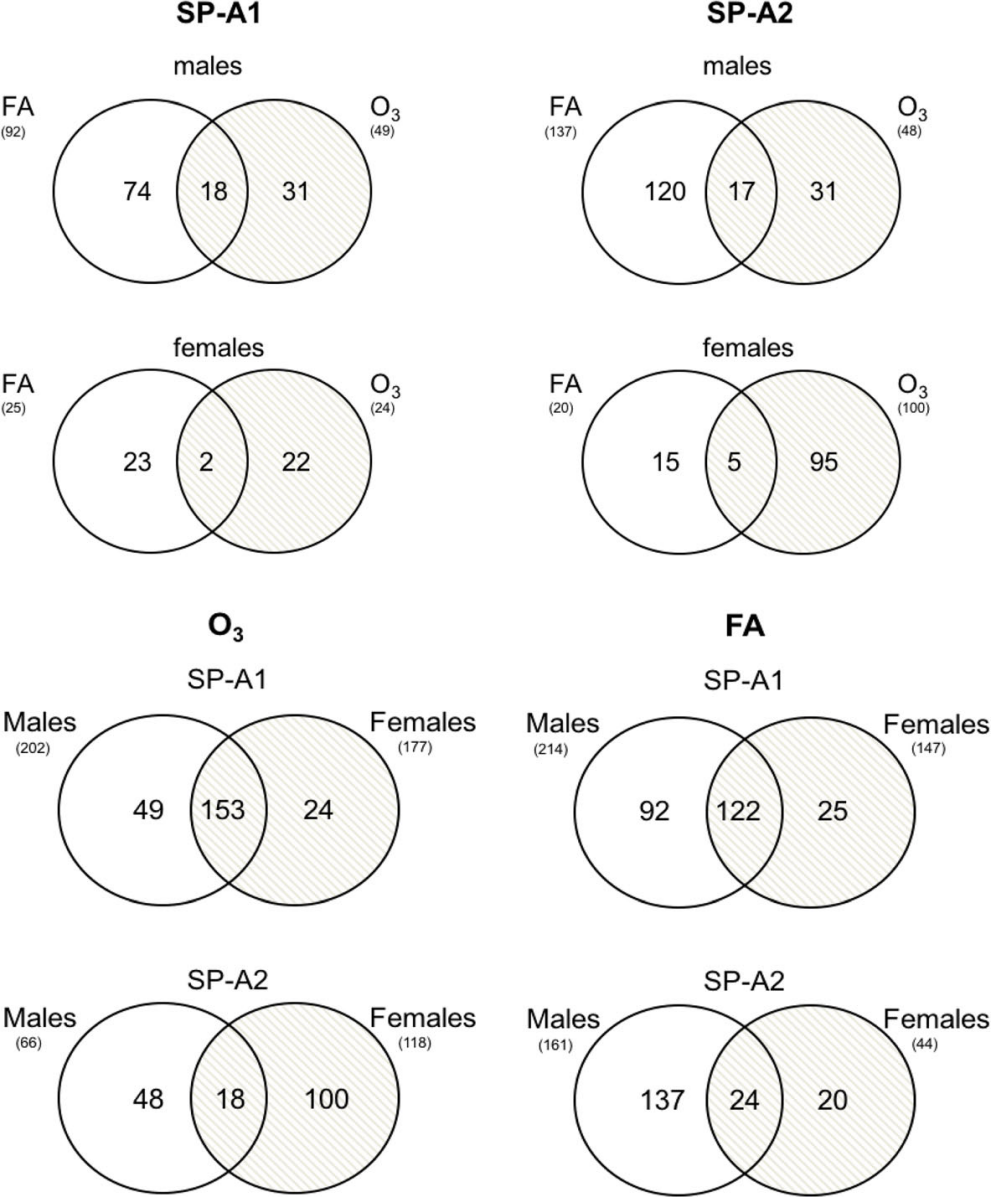
Venn diagrams showing the distribution of differentially expressed SP-A1 and SP-A2 AM miRNAs that changed ≥ 2-fold compared to SP-A KO AM, which are unique or common (overlapping areas) in FA and O_3_ exposure. Also, the figure shows the effect of sex on the expression of miRNAs after FA and O_3_ exposure. Venn diagrams are showing unique and commonly identified miRNAs among SP-A1 and SP-A2 male and female hTG mice. Out of the total 226 miRNAs identified in male and female SP-A1, 49 identified miRNAs are unique to male and 24 are unique to female; 153 miRNAs are identified in common after O_3_ exposure. In the case of SP-A2, out of the 166 miRNAs identified, 48 miRNAs are unique to male and 100 are unique to female; 18 miRNAs were identified in common after O_3_ exposure. However, under control conditions, more miRNAs (*n* = 122) were in common in the SP-A1 male and female mice than in the SP-A2 (*n* = 24)

Next, we focused on the AM miRNAs whose expression is altered in response to OxS from SP-A1 and SP-A2 male and female mice. We compared the SP-A1 and SP-A2 AM miRNome altered ≥ 2-fold between males and females under basal conditions and OxS (Fig. 3). We found that in SP-A1 mice, in males, 92 miRNAs were changed under control conditions and 49 under O_3_, whereas in females, only 25 miRNAs were changed in FA and 24 in OxS. The same comparison in SP-A2 AM miRNome showed that for males, 137 miRNAs were regulated in FA and 48 miRNAs in O_3_, while in females, only 20 were regulated in FA and 100 by OxS (Fig. 3).

### Sex- and gene-specific AM miRNAs that are regulated by OxS

To identify sex- and gene-specific AM miRNome changes in response to OxS, we compared via Venn diagrams the SP-A1- and SP-A2-regulated miRNAs that were altered in the two conditions (FA and O_3_) (Fig. 3 and Additional file 2). The diagrams show the distribution of the differentially expressed SP-A1 and SP-A2 miRNAs regulated ≥ 2-fold that are unique or common under basal conditions and under OxS in SP-A1 or SP-A2 male and female mice.

We next assessed the miRNAs unique and common for male and female SP-A1 and SP-A2 mice after O_3_ and FA exposure. Out of the total 372 miRNAs of our array, we identified 49 miRNAs that are unique to SP-A1 male and 24 miRNAs unique to SP-A1 females in OxS, and 153 miRNAs were found to be in common in SP-A1 males and females after O_3_ exposures (Fig. 3). In the case of SP-A2 mice, we identified 48 miRNAs unique to SP-A2 male and 100 miRNAs unique to SP-A2 females in OxS, and only 18 miRNAs were commonly identified after O_3_ exposure. Similarly, under control conditions in filtered air (FA), more miRNAs (*n* = 122) were in common in SP-A1 male and female mice than in SP-A2 (*n* = 24) (Fig. 3). These data indicate sex variability in the SP-A2 and SP-A1 miRNome.

### Ingenuity Pathway Analysis of the AM miRNome and the genes that they affect in response to OxS in SP-A2 male mice

To better understand and integrate the AM miRNome data, we performed Ingenuity Pathway Analysis (IPA) for the AM miRNAs whose expression was significantly altered by OxS. Only miRNAs that were shown to pass the corrected Bonferroni *p* < 0.0166 and an FDR-adjusted *q* < 0.05 were used to ensure that sex, treatment, gene, and array variability do not lead to false discoveries. IPA for the male SP-A2 miRNome (*n* = 31 miRNAs were male-specific) showed several mRNA transcripts, the expression of which could be affected by miRNAs that were changed by OxS. We found direct associations between some of these miRNAs with IL-6, NF-kB, STAT3, BCL2, and FOXO1 mRNAs.

miR-191-5p, which binds IL-6 [44], is downregulated. The levels of microRNAs (miR-21-5p, miR-181a-5p, miR-1195) in response to O_3_ are shown in Table 1; these are known to interact directly with STAT3.

**Table 1:** Levels and statistical significance of the male SP-A2 AM miRNAs compared to SP-A KO in OxS and shown by IPA to be directly associated with genes IL-6, NF-kB, STAT3, BCL2, and FOXO1

Mature ID	Fold regulation	*p* value
miR-191-5p	− 2.1515	0.00196
miR-153-3p	4.2526	0.000167
miR-15b-5p	− 1.7279	0.01395
miR-16-5p	− 1.8298	0.000532
miR-181a-5p	− 1.5004	0.000539
miR-183-5p	2.0782	0.000372
miR-195a-5p	− 1.2763	0.030639
miR-21a-5p	− 2.4629	0.009501
miR-9-5p	− 3.0084	0.001602
miR-486b-5p	1.407	0.196574
miR-1195	2.1028	0.000323

− indicates downregulation

We found five miRNAs to be directly associated with the BCL2 gene. Both miR-21-5p and miR-16-5p were significantly downregulated in our study. miR-195a-5p that has the same seeding sequence with miR-16-5p and is predicted to bind BCL2 mRNA was also downregulated while miR-153-3p that was also found to bind BCL2 experimentally (by Western blot, qRT-PCR, and LUC) [45] was increased.

Two miRNAs (miR-9-5p and miR-183-5p) were regulated by O_3_, and these were shown to target the NF-kB protein and mRNA experimentally (Western blot and qRT-PCR) [46] and by in silico analysis (TargetScan). miR-9-5p expression levels were downregulated. miR-183-5p, which was upregulated, probably has the opposite effect. FOXO1 is targeted by a multitude of miRNAs that are changed in our study miR-9-5p, miR-21-5p, miR-16-5p, miR-183-5p [47], miR-486b-5p, and miR-153-3p. The above six miRNAs were either up- or downregulated in our experiments (Table 1) and could potentially regulate the expression levels of FOXO1 in AM by OxS.

### Only the male SP-A2 AM miRNome in response to O_3_ associates with genes involved in OxS

When an IPA like the one done with data of male SP-A2 hTG mice was performed, using the 95 miRNAs found in the SP-A2 female AM miRNome, the main associations found were for cervical cancer and reproductive system disorders (data not shown). Although the SP-A2 females express the same miRNAs as the males, the changes resulting from O_3_ exposure are lower than those observed in the males, leading to the identification of different pathways. For the SP-A1-regulated miRNAs in males that changed in response to O_3_ (31 miRNAs), no major networks were identified, whereas for the SP-A1-regulated miRNAs in females (22 miRNAs), we found that tumor protein p53 may be involved in the regulation of 4 miRNAs identified in our study, along with argonaute-2 protein (AGO2), which is involved in biogenesis of miRNAs (data not shown).

### Effect of gonadectomy and OxS on expression of miRNAs in SP-A2 male and female mice

**Fig. 4:**
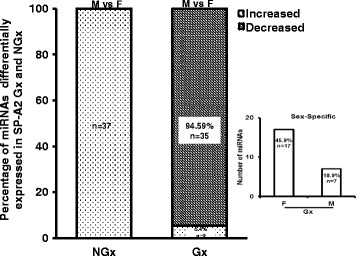
The effect of gonadectomy and OxS on AM miRNA expression profiles of SP-A2 mice. The differentially expressed miRNAs in SP-A2 non-gonadectomized (NGx) and gonadectomized (Gx) mice were identified after normalizing to corresponding NGx and Gx KO. NGx shows the miRNAs (*n* = 37) that changed significantly (≥ 2-fold) in OxS when males were compared to females. Gx depicts the comparison of Gx values (male vs female) to NGx (male vs female). Out of the same 37 miRNAs (found to have their levels increased in NGx), 2 miRNAs (5.4%) showed a significant increase (≥ 2-fold) and 35 miRNAs (94.59%) showed a significant decrease (≤ 2-fold). Inset depicts the comparison, of the 37 differentially expressed miRNAs, between Gx male and female. Out of 37 miRNAs studied, 17 miRNAs (45.9%) are significantly increased in female (≥ 2-fold), and 7 miRNAs (18.9%) are significantly increased in male (≥ 2-fold)

To identify the effect of sex hormones on the expression of miRNAs after OxS, we performed miRNA expression analysis in AM from gonadectomized (Gx) SP-A2 and KO male and female mice and compared it with that of non-gonadectomized (NGx) mice after O_3_ exposure. For this analysis, we used 63 miRNAs that were identified in both NGx (male and female) and Gx (male and female) groups. Of these, in the NGx (male vs female) group, only 37 miRNAs had their levels significantly changed (fold change ≥ 2) (Fig. 4, Additional file 3). In the Gx group (male vs female) compared to the corresponding NGx (male vs female) group, expression of 2 miRNAs (5.4%) was significantly increased (fold change ≥ 2), and expression of 34 miRNAs (94.6%) was significantly decreased (fold change ≤ 2). Of the 37 miRNAs differentially expressed in Gx male and female, 17 miRNAs (45.9%) are Gx female specific and 7 miRNAs (18.9%) are Gx male specific (Fig. 4 inset, Additional file 3). Moreover, a one-way ANOVA pertaining to the gonadectomy effect on the miRNA expression showed a significant difference with *F* stat = 11.98 with *F* crit = 3.92 and *p* = 0.00073 (see Additional file 3). These data indicate that sex hormones play an important role in the observed miRNA sex differences by affecting regulation of miRNAs, as most of the miRNAs that were increased in NGx were decreased in Gx.

### Expression of genes regulated by OxS in SP-A2 males

**Fig. 5:**
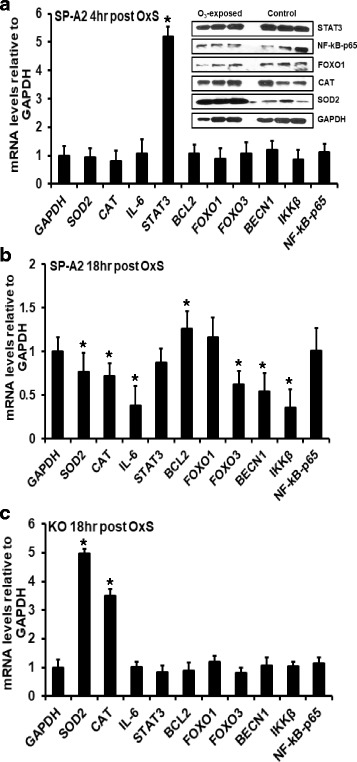
Effect of O_3_ in males. **a** mRNA levels of GAPDH, SOD2, CAT, IL-6, STAT3, BCL2, FOXO1, FOXO3, BECN1, IKKβ, and NF-kB-p65 genes were measured in AM of male SP-A2 mice 4 h after O_3_ exposure. mRNA levels were measured by qRT-PCR and normalized to GAPDH. STAT3 mRNA levels were significantly increased by 5-fold (*p* < 0.05), while the levels of other mRNAs did not change significantly compared to FA exposure. Inset depicts protein expression levels of STAT3, NF-kB-p65, FOXO1, CAT, and SOD2 in AM of male SP-A2 mice 4 h after O_3_ exposure. Protein levels were measured by Western blot analysis and normalized to GAPDH. **b** mRNA levels of the above mentioned genes were measured by qRT-PCR 18 h post exposure. In male SP-A2, O_3_ exposure significantly increased BCL2 mRNA levels compared to FA (*p* < 0.05). The mRNA levels of SOD2, CAT, IL-6, FOXO3, BECN1, and IKKβ were significantly decreased compared to FA (*p* < 0.05). **c** In KO, mRNA levels of SOD2 and CAT were significantly increased (*p* < 0.05) 18 h post exposure, while the levels of other mRNAs did not change significantly compared to FA. Data were generated using 3 animals/group, *n* = 18, and 3 replicate/animal

We performed qRT-PCR to assess the expression levels of SOD2, CAT, IL-6, STAT3, BCL2, FOXO1, FOXO3, BECN1, IKKβ, and NF-kB-p65 genes at mRNA level in the male SP-A2 AM. We found that under OxS, the levels of STAT3 mRNA were increased significantly (*p* < 0.05) by 5-fold (Fig. 5a). No significant differences were observed for the other mRNAs studied.

We examined the protein expression levels of STAT3, NF-kB, FOXO1, CAT, and SOD2 in the male SP-A2 AM after 4 h OxS. The Western blot analysis indicated no significant difference in the protein levels of STAT3, NF-kB, FOXO1, and CAT at 4 h after OxS exposure, whereas for SOD2, a significant increase was observed compared to control (Fig. 5a, inset).

To account for half-life differences and study further the expression of these genes, we used AM from male SP-A2 and KO mice after 18 h of exposure to O_3_ and found a significant increase in the mRNA level of BCL2 (Fig. 5b). The levels of the other genes studied (SOD2, CAT, IL-6, FOXO3, BECN1, and IKKβ) were decreased significantly (Fig. 5b). In contrast, in KO, the expression of SOD2 and CAT was increased significantly, but no significant changes in the expression of the other genes were observed (IL-6, STAT3, BCL2, FOXO3, BECN1, IKKβ, and NF-kB-p65 (Fig. 5c). A trend of increase in the level of FOXO1 was observed in SP-A2 and KO (Fig. 5b, c).

**Fig. 6:**
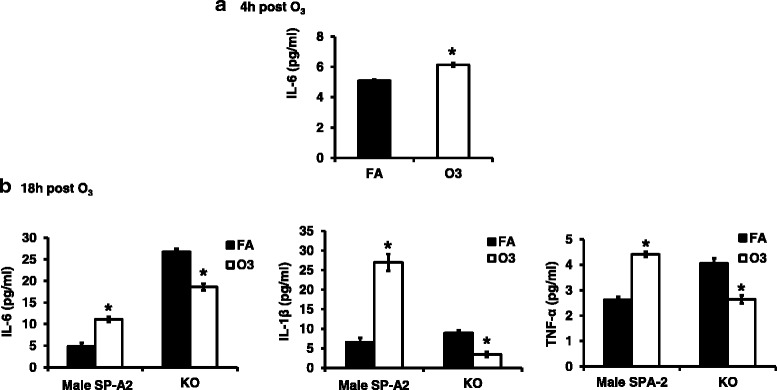
Effect of O_3_ on IL-6, TNF-α, and IL-1β protein levels in the bronchoalveolar lavage of male SP-A2 and KO mice compared to FA exposure. **a** Protein levels were measured by ELISA 4 h post exposure. OxS significantly increased IL-6 levels in SP-A2 male mice compared to FA (control) SP-A2 mice (*p* < 0.05). **b** Protein levels were measured at 18 h post exposure in SP-A2 and KO. OxS significantly increased IL-6, IL-1β, and TNF-α in SP-A2 mice (*p* < 0.05) and significantly decreased in KO mice (*p* < 0.05). Data were generated using 3 animals/group, *n* = 18 and 3 replicate/animal

To investigate the role of IL-6 in the increased STAT3 levels, we measured IL-6 protein by ELISA in the BAL of male SP-A2 hTG mice after exposure to O_3_ and FA. We found that IL-6 levels were increased significantly compared to control conditions (FA) at 4 h post OxS (Fig. 6a) and at 18 h post OxS (Fig. 6b). We also measured the levels of IL-1β and TNF-α at 18 h post OxS in SP-A2 and KO. We observed a significant increase in their levels in SP-A2 compared to FA (Fig. 6b), whereas in the case of KO, the level of these proteins was significantly decreased after O_3_ exposure compared to FA (Fig. 6b), indicating a role of SP-A2 in these processes.

### Apoptosis induced by OxS in male SP-A2 and KO mice

**Fig. 7:**
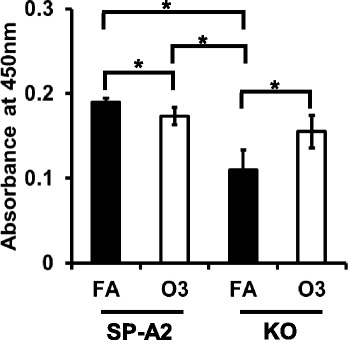
Effect of O_3_ on AM apoptosis by TUNEL assay. The TUNEL assay was performed at 18 h post exposure to ozone in SP-A2 and KO mice by using AM cells (70,000 cells/well). Data were generated using 3 animals/group (*n* = 12) and 4 replicate/animal (*p* < 0.05)

We used a TUNEL assay to assess apoptosis. We analyzed apoptotic cells from AM of SP-A2 and KO male mice at 18 h post OxS. In response to O_3_ exposure, no significant differences were observed between SP-A2 and KO. However, a small but significant decrease in apoptotic cells was observed in SP-A2 O_3_-exposed mice compared to SP-A2 FA-exposed mice, whereas an increase in apoptotic cells was observed in O_3_-exposed KO mice compared to FA-exposed KO (Fig. 7). Our results indicate that ozone differentially affects apoptosis in KO and SP-A2 AM, with SP-A2 providing perhaps some protection from the effect of OxS.

## Discussion

SP-A is an important host defense molecule. In humans, there are two SP-A functional genes, SFTPA1 (SP-A1) and SFTPA2 (SP-A2), and their gene products have been shown to differ functionally from each other [39, 48–50]. Differences have been seen in the proteomic expression of AM and the AM actin cytoskeleton after exposure to SP-A1 or SP-A2 variants [20, 48]. We have also previously shown sex differences in the KO AM proteome after treatment with SP-A1 or SP-A2 gene products where more changes occurred with SP-A1 and SP-A2 treatment in females and males, respectively [51]. The present study was done to further understand mechanisms underlying the previously observed sex differences in the AM function and proteome in response to SP-A genes (SP-A1 and SP-A2).

We studied the AM miRNome in male and female mice that express human SP-A1 and SP-A2 after O_3_ exposure and compared it to that of KO mice that do not express SP-A. In both male and female miRNomes, changes were found after exposure to O_3_ that may reflect an attempt to mitigate the consequences of OxS. In the male miRNome of both transgenic mice (SP-A1 and SP-A2), there was an increase in the number of miRNAs that changed significantly after O_3_ exposure, whereas in females, OxS did not change the miRNome substantially.

We found miR-191-5p, shown previously to regulate IL-6 [44], to be downregulated and this could increase the levels of IL-6, which is consistent with our experimental findings. IL-6 was found to be expressed in the BAL of males after O_3_ exposure, but not in females [38]. IL-6 has been shown to have both pro- and anti-inflammatory functions [52], and in mice, IL-6 overexpression has been shown to protect against hyperoxia-induced lung mitochondrial damage [53].

**Fig. 8:**
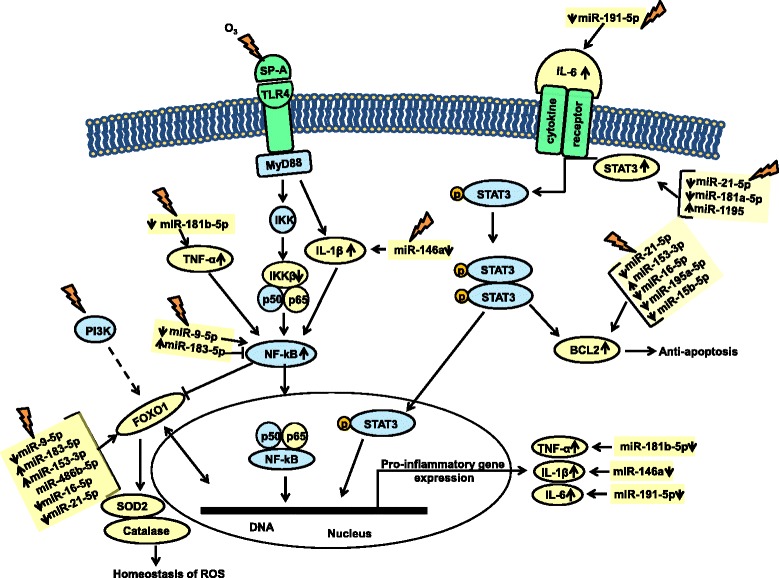
Schematic representation for the role of miRNAs in mediating regulation of pathways involved in anti-apoptotic, inflammatory, and reactive oxygen species on AM of SP-A2 mice compared to KO mice in response to O_3_. The miRNAs and genes studied in the present study are highlighted with yellow. Up (↑) and down (↓) arrows indicate increase or decrease

Pro-inflammatory cytokine IL-6 has been reported to activate STAT3 via phosphorylation [54–59] and OxS to trigger STAT3 phosphorylation that is responsible for transcriptional activation of cytokine-encoding genes that are involved in inflammation and cell injury [60]. miR-21-5p, miR-181a-5p, and miR-1195 shown in the present study to be altered significantly in response to O_3_ have also been shown to directly interact with STAT3. Specifically, miR-21-5p is predicted to bind STAT3 mRNA (TargetScan). In our experimental conditions, the observed downregulation of this miRNA is associated with increased levels of STAT3. However, activation of STAT3 by IL-6 has been shown experimentally to increase expression of miR-21-5p [61]. miR-1195 has been shown by in silico analysis to bind STAT3 [62], while miR-181a-5p has been shown with quantitative RT-PCR to be increased after activation of STAT3 by IL-6 [63]. In response to OxS, the expression levels of miR-21-5p, miR-1195, miR-181a-5p, and miR-191-5p (as shown in Fig. 8) were changed significantly and this could result in the observed increased levels of STAT3. We postulate that an activated or dimerized STAT3 translocates to the nucleus where transcription initiation of pro-inflammatory genes takes place (Fig. 8). Moreover, the significantly increased STAT3 mRNA levels and the increased levels of TNF-α, IL-6, and IL-1β after O_3_ exposure in the SP-A2 male AM (4 and 18 h post OxS) support this possibility. The IL-6 driven STAT3 phosphorylation and activation in OxS has been shown before [64] and has been linked with inflammation, cell injury, and cancer [65, 66], but the potential role of SP-A2 in this process is novel.

STAT3 activates other genes involved in cell cycle and upregulates anti-apoptotic genes such as BCL2 [67]. Of interest, BCL2 was found to be regulated by the male SP-A2 AM miRNome in OxS; BCL2 has been shown previously to be expressed in AM [68]. We found five SP-A2-regulated miRNAs that are directly associated with the BCL2 gene. miR-21-5p is predicted to bind both, BCL2 and STAT3 mRNAs (TargetScan), and miR-16-5p has been shown to bind BCL2 by several experimental approaches (Western blot, qRT-PCR, and luciferase reporter assays) [69, 70]. Both miR-21-5p and miR-16-5p were downregulated significantly in our study, and this is associated with increased mRNA levels of BCL2 (at 18 h post exposure, Fig. 5b) that may contribute to inhibition of apoptosis and cell proliferation in SP-A2 males, but not in KO males, where the expression of BCL2 did not change (Fig. 5c). miR-195a-5p that has the same seeding sequence with miR-16-5p, is predicted to bind BCL2 mRNA, and this was also downregulated in our study and may increase further the anti-apoptotic effects of miR-21-5p and miR-16-5p. miR-153-3p that binds BCL2 experimentally (by WB, qRT-PCR and LUC) [45] was increased in our study and may have the opposite effect, i.e., attenuating the anti-apoptotic effect of the other miRNAs. A further support for a role of BCL2 in the inhibition of apoptosis and cell proliferation in response to O_3_ is provided by the TUNEL assay. Fewer apoptotic cells were found in SP-A2 compared to FA and an increase in apoptotic cells in KO compared to FA (Fig. 7). Of interest, in the absence of SP-A (i.e., in KO mice), higher inflammation and lower survival rates were observed in both FA- and ozone-exposed mice compared to wild type animals [35, 40]. These findings together indicate that ozone differentially affects apoptotic pathways in SP-A2 and KO mice and that the lungs from SP-A2 mice may have a protective anti-apoptotic role.

Another transcriptional factor that we found to be targeted by the male SP-A2 AM miRNome and is involved in immune responses is NF-kB [71]. Two miRNAs (miR-9-5p and miR-183-5p) were significantly changed by O_3_, and these were shown to target the NF-kB mRNA experimentally [46] and by in silico analysis (TargetScan), respectively. miR-9-5p expression levels were downregulated significantly compared to control, and this may increase NF-kB mRNA levels that in turn may induce anti-proliferative effects [72]. miR-183-5p that was upregulated may have the opposite effect on NF-kB. Our data showed a decrease in IKKβ without any change in the NF-kB-p65 levels. It is well established that the IKKβ-dependent NF-kB signaling is a regulator of cell survival, immunity, and inflammation [73]. We know that SP-A activates NF-kB in THP-1 cells, a human macrophage-like cell line [74]. The signal may be induced through MyD88, IL-1β, and IKKβ kinase (IKK), with subsequent NF-kB translocation to the nucleus to facilitate transcription of pro-inflammatory genes, such as TNF-α and IL-1β as observed in the present study. We have observed that O_3_ exposure decreases the ability of SP-A to activate the NF-kB pathway as indicated by lack of increase of nuclear p65 and decrease of cytoplasmic IKBa [25]. However, no studies up to now have addressed the impact of O_3_ on SP-A2-mediated NF-kB activation.

The two transcriptional factors identified here, NF-kB and STAT3, are believed not to act in isolation but to cross talk with each other in inflammatory conditions [75]. It has been documented in the literature that IL-6 increases levels of NF-kB [76] and siRNA inhibition of IL-6 results in reduced levels of NF-kB [77]. Along with IL-6, TNF-α also increases the binding of NF-kB to STAT3 [78]. In our experimental conditions, 18 h post OxS, we observed an increase in TNF-α and IL-1β levels and 4 h post OxS we observed a decrease in miR-181-5p and miR-146a; these miRNAs are known to target TNF-α and IL-1β, respectively [79]. This may result in an increased level of TNF-α and IL-1β (Figs. 6b and 8). Moreover, an increased level of TNF-α and IL-1β may activate NF-kB, causing its translocation to the nucleus to facilitate transcription of pro-inflammatory genes as discussed above. Furthermore, an activated NF-kB has been shown to increase activation of BCL2 [80], whereas a mutant NF-kB decreased BCL2 [81]. The present data indicate that SP-A2 may play a role in these processes in the male AM miRNome in response to OxS.

Another transcript in our study that was targeted by several miRNAs is the FOXO1. This mRNA is targeted by miR-9-5p [47], miR-21-5p, miR-16-5p (TargetScan), miR-183-5p [47], miR-486b-5p [82], and miR-153-3p [47]. From the above six miRNAs, all but miR-486b-5p were changed significantly in our study and these potentially could regulate the expression levels of FOXO1 in AM under OxS (Fig. 8). FOXOs are transcription factors known to be involved in the homeostasis of ROS and can function as a negative feedback loop to control cellular reactive oxygen species [83]. FOXO1 regulates the expression of anti-oxidant genes such as CAT and SOD2, both of which are known to neutralize free radicals generated by ROS. Although no significant changes were observed in FOXO1 mRNA levels at 4 or 18 h post OxS in either SP-A2 or KO mice, the mRNA levels of SOD2 (superoxide dismutase) and CAT (catalase) significantly decreased at 18 h post OxS in SP-A2 males. However, for SOD2 (but not for CAT) at 4 h post OxS, the protein levels were increased. In contrast, in KO males, the mRNA levels of SOD2 and CAT were significantly increased. This striking difference between the KO and the hTG indicates that the SP-A2 gene may play a role in the homeostasis of ROS.

Apart from the miRNAs that affect the expression of FOXO1, activated NF-kB through TNF-α and IL-1β may repress the expression of FOXO1. This in turn causes inhibition of FOXO1-mediated anti-oxidant defense mechanism at post-transcriptional level during OxS in AM. Our previous studies have shown that SP-A may prime AM to be ready to respond to oxidative stress. Moreover, because SP-A is shown to be oxidized more readily than other proteins after ozone exposure [40], we had postulated that SP-A may serve as an anti-oxidant “sacrificial lamb” by scavenging ROS and thus protect other molecules from immediate oxidative damage [40]. In the absence of SP-A, there may be an increased level of oxidative stress and this may compromise the function of AM [35]. Furthermore, the AM expression profile of SP-A2 mice was most similar to that of WT mice, particularly for proteins regulated by Nrf2, which were downregulated compared to KO, suggesting that these proteins were utilized to ameliorate the effect of oxidative stress in SP-A2 [9, 48], by OxS.

## Conclusions

In summary, SP-A2 regulates miRNAs that play a role in pathways involved in inflammation, apoptosis, and ROS homeostasis. The observations made pertain only to the male SP-A2 AM miRNome in response to OxS exposure and not to females. The involvement of sex hormones in the observed miRNA sex-specific differences was supported by the findings in gonadectomized mice where we observed that the regulation of the miRNome of the SP-A2 male mice compared to that of female mice in response to OxS is significantly altered after gonadectomy. Collectively, our findings support and in part explain our previous observations of sex differences after OxS, where we observed females to be more susceptible in terms of survival to OxS than males. Here, we show that the male SP-A2 AM miRNome in response to OxS is associated with OxS-related inflammatory genes, regulation of ROS, and apoptosis. In females, although these miRNAs are expressed, their levels do not change significantly. The miRNAs identified here may correlate with the male survival advantage in OxS compared to females.

## Additional files

Additional file 1: Original miRNA data used to generate Figs. 1 and 2. (XLSX 1320 kb)

Additional file 2: Original miRNA data used to generate Venn Diagrams (Fig. 3). (XLSX 75 kb)

Additional file 3: Original gonadectomy miRNA tag count data (Fig. 4). (XLSX 123 kb)

## Abbreviations

AM: Alveolar macrophage
ANOVA: Analysis of variance
BCL2: B cell lymphoma 2
BECN1: Beclin-1
CAT: Catalase
*F* crit: Critical value in *F* distribution
*F* stat: *F* test or Fisher’s statistic
FA: Filter air
FDR: Benjamini and Hochberg false discovery rate
FOXO1: Forkhead box O1
FOXO3: Forkhead box O3
Gx: Gonadectomized
hTG: Humanized transgenic mice
IKKβ: Inhibitor of nuclear factor kappa-B kinase subunit beta
IL-1β: Interleukin 1 beta
IL-6: Interleukin 6
KO: Knockout
miRNA: Micro-RNA
NF-kB: Nuclear factor kappa-light-chain-enhancer of activated B cells
NGx: Non-gonadectomized
O_3_: Ozone
OxS: Oxidative stress
qRT-PCR: Quantitative real-time polymerase chain reaction
SFTP-A1: Gene encoding SP-A1
SFTP-A2: Gene encoding SP-A2
SOD2: Superoxide dismutase 2
SP-A: Surfactant protein A
STAT3: Signal transducer and activator of transcription 3
TNF-α: Tumor necrosis factor alpha
TUNEL: TdT-mediated dUTP Nick-End Labeling
